# Integrated Host Genetics and Skin Microbiome Profiling Suggest an HLA-C–Peptostreptococcus Axis in Psoriasis

**DOI:** 10.3390/ijms27094116

**Published:** 2026-05-04

**Authors:** Oliver Seifert, Malin Assarsson, Lokeshwaran Manoharan, Jan Söderman

**Affiliations:** 1 Department of Clinical and Experimental Medicine, Faculty of Health Sciences, Linköping University, S-581 83 Linköping, Sweden; 2 Division of Dermatology and Venereology, Region Jönköping County, S-551 85 Jönköping, Sweden; 3 National Bioinformatics Infrastructure Sweden (NBIS), SciLifeLab, Division of Occupational and Environmental Medicine, Department of Laboratory Medicine, Lund University, S-223 81 Lund, Sweden; 4 Laboratory Medicine, Region Jönköping County, S-551 85 Jönköping, Sweden

**Keywords:** psoriasis, skin microbiome, single-nucleotide polymorphisms (SNPs), HLA-C susceptibility locus, integrative multi-omics analysis

## Abstract

Psoriasis is a chronic immune-mediated disease driven by genetic susceptibility and environmental factors, including microbial exposure. While HLA-C-linked variants represent the strongest genetic risk factors, their relationship with the cutaneous microbiome remains incompletely understood. This study aimed to investigate host–microbiome interactions in psoriasis through integrative multi-omics analysis. Skin microbiome profiling using 16S rRNA sequencing and targeted genotyping of psoriasis-associated single-nucleotide polymorphisms (SNPs) was performed in lesional and non-lesional skin from patients with plaque psoriasis and in healthy controls. Integrated analysis was conducted using supervised multivariate modeling (DIABLO) to identify coordinated genetic and microbial features associated with disease status. Combined genetic and microbial signatures differentiated lesional, non-lesional, and healthy skin. Variants within the HLA-C susceptibility region, including rs12191877, rs10484554, and rs4406273, showed contributions to group separation and demonstrated positive associations with *Peptostreptococcus anaerobius*. Associations involving ERAP1 variants linked antigen-processing pathways with inflammation-associated microbial taxa in lesional skin. Importantly, genotype–microbiome correlations were also detected in clinically non-lesional skin, where an increased psoriasis risk allele dosage co-varied with a higher relative abundance of *P. anaerobius* and *Aerococcus urinae*. In contrast, commensal-associated taxa were enriched in healthy controls and formed genotype-linked clusters only in non-lesional skin. These findings suggest that psoriasis is characterized by coordinated host genetic and microbial interaction patterns centered on antigen presentation pathways. The presence of a genotype–microbiome coupling in non-lesional skin may indicate that genetically determined immune configurations could shape microbial community structure prior to visible lesion development. Rather than reflecting uniform dysbiosis, psoriasis may represent a dynamic host–microbe ecosystem in which genetic susceptibility influences microbial persistence and inflammatory readiness.

## 1. Introduction

Psoriasis is a chronic immune-mediated inflammatory skin disease affecting approximately 2–3% of the population [1]. Although classically regarded as a cutaneous disorder, psoriasis is now established as a systemic inflammatory condition associated with increased risks of metabolic syndrome, cardiovascular disease, inflammatory bowel disease (IBD), obesity and psychiatric comorbidities [2,3].

Psoriasis arises from a complex interplay between genetic susceptibility, environmental triggers, and immune dysregulation, leading to aberrant activation of the IL-23/Th17 axis and disrupted epithelial barrier function [4]. Emerging evidence suggests that microbial dysbiosis plays a role in psoriasis pathogenesis [5,6] and microbial colonization can trigger or exacerbate psoriatic inflammation, and shifts in microbiota composition have been reported across skin, gut, and oropharyngeal compartments [7,8,9].

Skin dysbiosis in genetically predisposed individuals can trigger activation of the innate immune system, which subsequently drives an adaptive immune response and may contribute to the development of psoriasis [10,11]. Streptococcal infections of the upper respiratory tract are the best-established bacterial trigger for psoriasis, particularly associated with the onset of guttate psoriasis [12,13,14,15]. For example, *Streptococcus pyogenes* infection is strongly implicated in psoriasis and can precipitate flares. Mechanistic studies suggest that streptococcal antigens drive expansion of cutaneous-homing CLA+ T cells and Th17 responses [16,17], while *Streptococcal* superantigens can bypass classical antigen presentation pathways and provoke broad T-cell activation [18]. Nonetheless, epidemiological evidence remains inconclusive, with a recent Cochrane review finding insufficient proof of a causal relationship between psoriasis and Streptococcal infection [19].

Studies of the psoriatic skin microbiome have yielded conflicting results, reporting both increased and decreased alpha-diversity in lesional skin relative to uninvolved skin or controls [11,20]. Specific taxa, including *Streptococcus*, *Staphylococcus*, and *Corynebacterium*, have been reported as enriched in psoriatic plaques, while commensal *Cutibacterium acnes* often appears reduced [21,22,23]. Our research group demonstrated that narrowband UVB therapy modulates the skin microbiota and that the relative abundance of several genera correlates with disease severity [24,25]. Together, these findings suggest that microbial alterations in psoriasis are subtle yet biologically relevant and may interact with host immune pathways.

Genetic susceptibility is a major determinant of psoriasis risk, with over 80 associated loci identified via genome-wide association studies [26]. The strongest association lies in the major histocompatibility complex (MHC) region, particularly HLA-C*06:02, which confers increased risk and is linked to a younger age of onset and guttate phenotype [27,28]. Additional susceptibility genes implicate pathways in antigen presentation, NF-kB signaling, interferon responses, and barrier function [27,28,29].

Increasingly, psoriasis is conceptualized as an immune disorder in which genetic predisposition influences host–microbe interactions. HLA alleles shape the presentation of microbial antigens and skin bacterial composition, suggesting an interplay between genetic background and microbial exposure [30]. However, few studies have simultaneously examined microbiome profiles and host genomic variation in psoriasis. Systems-level approaches integrating genomics and microbiome data offer an opportunity to understand how genetic susceptibility modulates microbial colonization and how this, in turn, affects disease phenotype. To date, integrated host–microbiome analyses in psoriasis remain scarce, and the mechanisms linking genetic variation to cutaneous microbial ecology are poorly understood.

The aim of the present study was to investigate whether psoriasis-associated genetic variants are associated with alterations in the cutaneous microbiome, and to determine whether a combined signature of specific skin microbiota and psoriasis-associated genetic variants can discriminate between phenotypic groups.

## 2. Results

### 2.1. Study Population

From the 68 included control samples and 39 samples from each non-lesional or lesional psoriatic skin (in total, representing 40 cases), two controls were removed as they were outliers, and two cases and one control were removed due to limited data regarding SNPs. Consequently, the final dataset used for the DIABLO analyses comprised 65 healthy controls, 37 lesional psoriasis samples, and 35 non-lesional psoriasis samples. Patients’ demographic and clinical characteristics are shown in Table 1.

For the lesional skin integration, the samples were first filtered to retain only those with both SNP and lesional microbiome data available. In a second filtering step, low-abundance microbiome features were removed, resulting in 65 control samples and 37 lesional psoriasis samples included in the integrative analysis.

Similarly, for the non-lesional skin integration, samples were initially filtered to include only those with both SNP and non-lesional microbiome data, followed by removal of low-abundance microbiome features. This yielded 65 control samples and 35 non-lesional psoriasis samples.

The raw microbiome dataset consisted of 26,968 amplicon sequence variants (ASVs). After quality control and abundance filtering, 824 ASVs remained. Taxonomic agglomeration to the species level using tax_glom() resulted in 313 bacterial species for the lesional versus control integration and 312 species for the non-lesional versus control integration.

### 2.2. Integrated Multi-Omics Analysis Separates Psoriatic and Healthy Skin Across Both Microbiome and SNP Data Blocks

**Figure 1:**
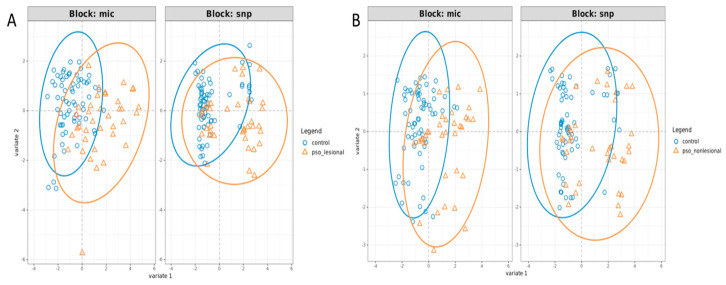
Integrated microbiome and SNP profiles distinguish psoriatic lesional and non-lesional skin from healthy controls. Psoriatic samples are shifted relative to controls along the first component across both data blocks, indicating coordinated genetic and microbial contributions to group separation despite partial overlap. DIABLO sample plots showing the first two latent components for microbiome (block: mic) and SNP (block: snp) data blocks comparing (**A**) lesional and (**B**) non-lesional skin with controls. Points represent individual samples; ellipses indicate 95% confidence intervals for group means (centroids).

The final DIABLO model integrating the microbiome (species level) and the SNP datasets revealed a separation between psoriatic lesional, non-lesional and control samples (Figure 1A,B). In both the microbiome block and the SNP block, and for both group comparisons (i.e., control samples versus either lesional or non-lesional psoriasis samples), the psoriasis samples were shifted toward positive values along the first latent component, and the control samples toward negative values. Confidence ellipses indicated that the groups were not completely separated, but there were directional shifts across both data blocks and sample types.

### 2.3. HLA-C–Linked Variants and Specific Microbial Taxa Drive Discrimination Between Psoriasis and Controls

**Figure 2:**
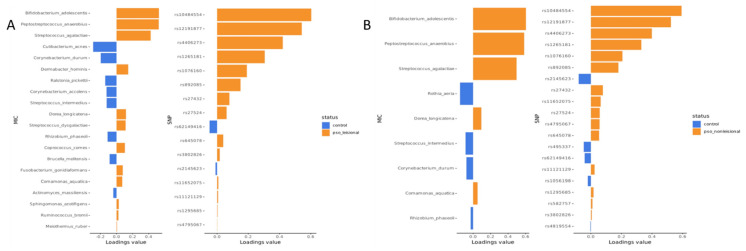
HLA-C-linked SNPs and inflammation-associated microbial taxa drive discrimination between psoriasis and controls. Feature-loading plots for the first latent component from the DIABLO analysis showing the most discriminant microbiome (MIC) and SNP features for (**A**) lesional psoriasis vs. controls and (**B**) non-lesional psoriasis vs. controls. Bar colors indicate the group with a higher relative abundance (microbiome) or allele dosage (SNPs). Several microbial taxa, including *Peptostreptococcus anaerobius*, *Streptococcus agalactiae*, and *Bifidobacterium adolescentis*, show higher contributions in psoriatic samples, whereas taxa such as *Corynebacterium durum* and *Streptococcus intermedius* are associated with controls. Within the SNP block, variants located in or near the HLA-C susceptibility region (including rs10484554, rs12191877, and rs4406273) show the strongest contributions to group separation.

Feature loading plots for the first DIABLO component (Figure 2A,B) identified the microbial species and SNP markers contributing most strongly to the latent components, and, in extension, to group separation and classification of psoriatic lesional, non-lesional and control samples. Several taxa were major contributors to the discrimination. Species such as *Bifidobacterium adolescentis*, *Peptostreptococcus anaerobius* and *Streptococcus agalactiae* had positive loading on the first latent component and showed a higher abundance in psoriatic lesional and non-lesional samples compared with skin from healthy individuals. Species with a negative loading included *Corynebacterium durum* and *Streptococcus intermedius*, with higher abundance in control samples compared with both lesional skin and non-lesional skin, whereas, e.g., *Cutibacterium acnes* and *Rothia aeria,* with negative loading, showed higher abundance in lesional and non-lesional samples, respectively.

Within the SNP block, several loci showed positive loading, most notably rs10484554, rs12191877, rs4406273 and rs1265181, all located in or near the HLA-C region and PSORS1C3 on chromosome 6. These variants were strongly associated with the psoriatic lesional and non-lesional groups. Also, rs1076160, located within the TSC1 gene on chromosome 9, showed a positive loading with higher allele dosage associated with the psoriatic lesional and non-lesional groups. In contrast, rs2145623 and rs62149416 showed negative loading with a low overall loading strength in lesional and non-lesional skin.

### 2.4. Peptostreptococcus anaerobius Shows Associations with HLA-C Risk Variants

**Figure 3:**
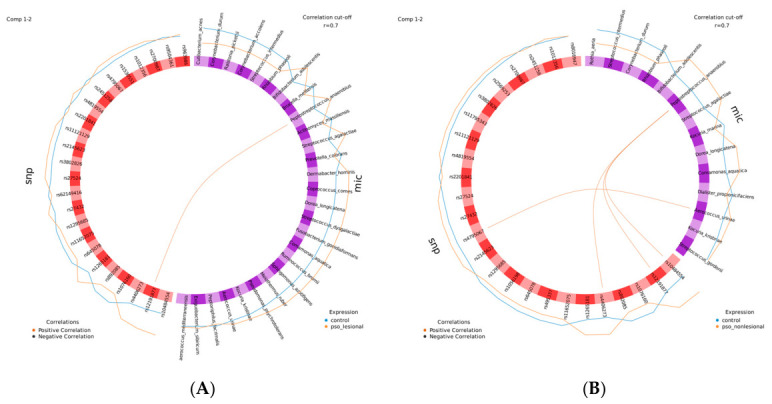
*Peptostreptococcus anaerobius* shows associations with HLA-C risk variants in lesional and non-lesional psoriasis. Circos plots visualize correlations between discriminant features from the DIABLO models for lesional (**A**) and non-lesional (**B**) analyses. In lesional skin, a strong positive association is observed between rs12191877 and *Peptostreptococcus anaerobius*. In non-lesional skin, *P. anaerobius* shows positive correlations with multiple HLA-C-linked variants (rs4406273, rs12191877, and rs10484554), and additional associations are observed with *Aerococcus urinae*. Genotypes (snp) are shown in red and microbiome (mic) features in purple. Correlation (cutoff 0.7) links are color-coded (brown = positive; grey = negative). Surrounding lines show the abundance/allele dosage of the selected variables for each sample type.

To explore cross-omics relationships between host genetic variants and the microbiome, Circos plots were generated from the final DIABLO models in order to visualize correlations between discriminant features from the DIABLO models (Figure 3). For the model based on psoriasis lesional skin versus skin from healthy controls, a single strong positive correlation was detected between SNP rs12191877 and *Peptostreptococcus anaerobius* (Figure 3A).

The Circos plot of the non-lesional DIABLO model (Figure 3B) revealed strong positive correlations between important SNPs and microbial taxa. Specifically, *Peptostreptococcus anaerobius* was positively correlated with three SNPs (rs4406273, rs12191877, and rs10484554), while *Aerococcus urinae* showed a positive correlation with rs4795067.

### 2.5. Integrated Heatmaps Reveal Host–Microbe Clustering and Heterogeneity in Psoriasis

**Figure 4:**
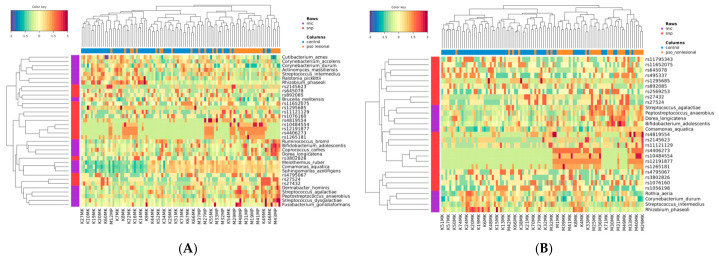
Integrated heatmaps reveal host–microbe clustering and heterogeneity in psoriasis. Clustered heatmaps showing co-variation between selected microbial species (mic, purple) and SNPs (snp, red) across (**A**) lesional vs. control samples and (**B**) non-lesional vs. control samples. Rows represent features, and columns represent samples. The heatmap color intensity represents standardized abundance values (based on either CLR-transformed values for species or allele dosage for SNPs), trimmed to a standard deviation interval of 3. Distinct clustering patterns separate psoriatic and control samples, with enrichment of taxa such as *Peptostreptococcus anaerobius* and *Streptococcus agalactiae*, alongside an increased allele dosage of HLA-C-linked SNPs in psoriatic samples. However, substantial heterogeneity is observed within psoriatic groups, indicating that host–microbe interaction patterns vary between individuals. Similar clustering trends in non-lesional skin further support the presence of genotype-associated microbial configurations beyond clinically visible lesions.

Clustered heatmaps (Figure 4) were generated to visualize the relationships between features (microbial species and SNPs selected by the final DIABLO model) and samples.

Clustering patterns were observed between psoriatic lesional and control groups; however, substantial heterogeneity remained within psoriatic samples, even among samples clustering closely together (Figure 4A). Especially *Peptostreptococcus anaerobius, Streptococcus agalactiae* and –*dysgalactiae* among species, as well as rs10484554, rs12191877, rs11121129, rs4406273 and rs1265181 located near HLA-C and PSORS1C3 among SNPs, seemed to more consistently show higher values among lesional samples. Notably, within the lesional cluster, samples exhibiting lower values at these HLA-C-proximal SNPs tended to show higher values for rs27524 and rs27432.

Clustering was also evident when comparing psoriatic non-lesional skin with control samples (Figure 4B). Non-lesional samples displayed enrichment of *Streptococcus agalactiae*, *Peptostreptococcus anaerobius*, *Dorea longicatena*, and *Bifidobacterium adolescentis.* Notably, *Streptococcus agalactiae, Peptostreptococcus anaerobius*, and *Bifidobacterium adolescentis* were also enriched in lesional skin. Non-lesional samples were further grouped according to genotypic variation at rs11121129, rs4406273, rs10484554, rs12191877, and rs1265181, all of which were also present in relation to lesional skin. Similar to lesional skin, these patterns were not uniform across all samples, underscoring heterogeneity within the non-lesional group.

## 3. Discussion

Psoriasis is increasingly understood as a complex immune-mediated disorder arising from the interplay between host genetic susceptibility, environmental exposures, and immune dysregulation. In this integrative multi-omics study combining host genetic variation with skin microbiome profiling, we found host–microbe patterns distinguishing lesional psoriasis, non-lesional psoriasis, and healthy skin. Beyond lesion-specific alterations, our findings suggest that psoriasis is associated with genotype-linked microbial configurations detectable in clinically unaffected skin, supporting the concept that non-lesional skin represents a pre-inflammatory state rather than unaffected tissue.

A major finding of this study was the contribution of variants located in or near the HLA-C susceptibility region to the separation of psoriatic and control samples, together with microbial taxa such as *Peptostreptococcus anaerobius.* These findings are in line with the well-established role of HLA-C in psoriasis susceptibility and immune regulation [31,32,33,34]. The repeated co-occurrence of HLA-C-linked variants with *P. anaerobius* across analyses suggests that antigen presentation pathways may be associated with differences in microbial community structure. Given the central role of HLA-C in CD8^+^ T-cell activation and immune surveillance [32,33,34,35,36,37,38], it is possible that genetically determined differences in antigen presentation influence the cutaneous immune environment in ways that favor the persistence of certain microbial taxa. However, these associations should be interpreted cautiously, as the present study does not establish causal or mechanistic relationships.

To our knowledge, associations between *P. anaerobius* and HLA-C-linked variants have not previously been reported in psoriasis. The observed co-variation between HLA-C-linked variants and *P. anaerobius* may be interpreted in the context of antigen presentation pathways central to psoriasis pathogenesis. HLA-C plays a key role in presenting peptides to CD8^+^ T cells, and variation within this locus has been associated with altered immune activation, disease phenotype, and responsiveness to environmental triggers [39]. Differences in HLA-C-mediated antigen presentation may, therefore, influence the repertoire of microbial- or self-derived peptides presented at the skin surface, potentially contributing to variation in local immune responses. In psoriasis, antimicrobial peptides such as LL-37 can function as autoantigens when presented via HLA class I molecules, leading to activation of autoreactive T cells [40]. It is, therefore, plausible that microbial components, including those derived from Gram-positive bacteria such as *P. anaerobius*, may be associated with antigen presentation processes, either by providing structurally relevant ligands or by enhancing local innate immune activation. Interactions between LL-37 and Gram-positive bacterial cell wall components, including those present in *P. anaerobius*, may enhance local inflammatory responses [41].

An alternative interpretation is that *P. anaerobius* may act primarily as an inflammatory amplifier rather than as a source of specific antigens. Previous studies have shown that *P. anaerobius* can activate innate immune pathways, including TLR2/4 signaling and downstream NF-κB and NLRP3 inflammasome activation, leading to increased production of pro-inflammatory cytokines such as IL-1β. Inflammasome-derived cytokines, including IL-1β and IL-18, are known to promote IL-17-mediated immune responses, which are central to psoriasis pathogenesis. Within this framework, the association between *P. anaerobius* and psoriasis may reflect its capacity to enhance local inflammatory signaling rather than direct involvement in antigen presentation. Such a mechanism would be consistent with the observed co-variation between microbial taxa and host genetic variants, without implying a direct antigen-specific interaction [42,43].

Interestingly, genotype–microbiome associations were not restricted to lesional skin but were also observed in non-lesional skin. An increased dosage of psoriasis risk alleles and variants in NOS2 (rs4795067) co-varied with higher relative abundance of *P. anaerobius* and *Aerococcus urinae*, suggesting that genetically influenced immune differences may shape microbial colonization prior to overt inflammation. Both taxa have been reported to activate innate immune signaling pathways, including Toll-like receptor-mediated responses and pro-inflammatory cytokine production [42,44].

In non-lesional skin of psoriasis patients, we observed a commensal-associated microbial cluster comprising *Rothia aeria*, *Corynebacterium durum*, *Streptococcus intermedius*, and *Rhizobium phaseoli* [45], which co-segregated with specific host genetic variants (rs3802826, rs1076160, rs1056198 and rs4795067). Notably, this association was absent in lesional skin. Functional annotation indicated that these variants localize to genomic regions involved in immune regulation, supporting a possible role for host genetics in shaping microbial community composition under non-inflammatory conditions. Rs1056198 maps to RNF114, a well-established psoriasis susceptibility locus involved in pathways known to influence host–microbe interactions at epithelial surfaces [46]. The intronic variant rs3802826 lies within ETS1, a transcription factor that regulates lymphocyte differentiation and immune activation, including mechanisms relevant to autoimmune and inflammatory skin disease [47]. Furthermore, rs1076160 is located in proximity to TSC1, a regulator of mTOR signaling implicated in epidermal differentiation, barrier integrity, and cellular metabolic control, processes that may shape microbial niche selection in the skin microenvironment [48].

In addition to HLA-C-linked variants, we observed associations involving loci related to antigen processing and immune regulation, including ERAP1 (rs27524 and rs27432) and NOS2 (rs4795067). ERAP1-mediated peptide processing influences the repertoire of peptides presented by HLA class I molecules, and epistatic effects between ERAP1 variants and HLA-C*06:02 have been reported in multiple immune-mediated inflammatory diseases [49,50]. One potential interpretation of our clustering results is that ERAP1-mediated variation in antigen processing may influence host immune responses to microbial-derived peptides, thereby contributing to genotype-dependent microbial structuring within inflamed skin.

Notably, the clustering pattern suggests that lesional samples with a relatively lower allele dosage at SNPs located near HLA-C tended to show a higher dosage at ERAP1 variants, proposing genetic effects rather than independent signals. Such reciprocal patterns may be consistent with models in which antigen-processing variation modulates the peptide repertoire available for presentation by HLA class I molecules, potentially altering immune recognition of microbial antigens during active inflammation.

Variation at the NOS2 locus encoding inducible nitric oxide synthase (iNOS) further supports this interpretation. NOS2 has been identified as a psoriasis susceptibility locus in genome-wide association studies and is closely linked to cutaneous inflammatory signaling [27]. Nitric oxide contributes both to immune regulation and to antimicrobial defense at epithelial surfaces, and recent experimental work suggests that even low levels of NOS2-derived nitric oxide may promote psoriasis-like inflammation [51]. Given its antimicrobial activity at the skin surface, NOS2 represents a possible mechanistic bridge between host genotype and microbial ecology, whereby genetically determined differences in nitric oxide signaling influence microbial persistence.

Microbial features contributing to discrimination between psoriatic and control skin included enrichment of *P. anaerobius*, *Streptococcus agalactiae*, and *Bifidobacterium adolescentis* in psoriatic samples, whereas commensal-associated taxa such as *Corynebacterium durum* and *Streptococcus intermedius* were more abundant in healthy controls. *B. adolescentis* has Th17-inducing, disease-exacerbating potential in autoimmune models and susceptible hosts [52]. Peptides derived from *S. agalactiae* can strongly activate autoreactive CD8^+^ T cells from psoriasis patients via a melanocyte-directed T-cell receptor recognizing the psoriasis autoantigen ADAMTSL5 [53]. Several *Corynebacterium* taxa, including *C. durum*, have been suggested to exert protective or homeostatic effects on the skin. Reductions in *Corynebacterium* have been reported in inflammatory skin diseases, and their abundance has been shown to increase alongside microbial diversity following effective therapy, supporting a potential beneficial role for these taxa in maintaining cutaneous immune balance [54]. Rather than indicating simple overgrowth of pathogenic organisms, these findings may indicate ecological imbalance involving enrichment of immunostimulatory taxa alongside depletion of potentially homeostatic commensals [11,21,22,23]. The enrichment of several taxa across both lesional and non-lesional skin suggests that psoriasis-associated microbial alterations are not restricted to plaques but may reflect systemic or genetically conditioned ecological shifts.

Substantial heterogeneity within psoriatic samples was evident despite group-level separation in multivariate models. This variability likely reflects interindividual differences in genetic background, immune activation, environmental exposure, or disease stage. The observed clustering patterns, therefore, suggest that multiple host–microbe interaction modules may underlie clinically similar phenotypes, potentially contributing to variability in disease expression and treatment response.

The strengths of this study include matched sampling of lesional and non-lesional skin, integration of genomic and microbiome datasets obtained from the same individuals, and application of supervised multi-omics modeling to identify coordinated cross-omics relationships. A key limitation of this study is the modest sample size relative to the complexity of multi-omics integration. Although the DIABLO framework reduces dimensionality and identifies coordinated patterns across data types, the number of samples limits statistical power and may affect model stability and generalizability. The present study should, therefore, be regarded as exploratory and hypothesis-generating. The identified genotype–microbiome associations, particularly those observed in non-lesional skin, require validation in independent and ideally longitudinal cohorts. Differences in sex distribution between cases and controls, as well as variability in disease severity, as reflected by PASI scores, may have influenced the observed microbial and genetic patterns and represent potential sources of residual confounding. However, the alpha- and beta-diversity of the microbiomes were analyzed and correlated with different patient factors, such as sex and age, and no significant correlation indicating potential confounding was found. In addition, although all patients were diagnosed with plaque psoriasis, heterogeneity in disease expression may contribute to variability within the psoriatic group.

The limitations further include the potential risk of model overfitting and the cross-sectional design, which precludes inference of causality or temporal relationships. Importantly, DIABLO loadings reflect the relative contribution to multivariate discrimination rather than independent biological effects, and the identified associations should, therefore, be interpreted cautiously. Several limitations related to the use of 16S rRNA sequencing should be considered. This approach provides limited taxonomic resolution compared with shotgun metagenomics and does not allow reliable discrimination at the strain level, which may be biologically relevant. In addition, 16S-based profiling is sensitive to primer selection and amplification biases, which can influence the detected microbial composition. The method also does not provide direct information on functional activity or metabolic capacity, nor does it distinguish between viable and non-viable bacteria. These limitations should be taken into account when interpreting associations between specific taxa and host genetic variation.

Longitudinal studies will be required to determine whether genotype–microbiome relationships precede disease flares or predict therapeutic response. Functional experiments examining immune responses to genotype-associated microbial taxa may further clarify the biological relevance of the observed correlations.

The findings of this study should be interpreted with caution. The identified associations between host genetic variants and microbial taxa are based on multivariate modelling and reflect patterns of co-variation rather than evidence of direct mechanistic interaction. While these results are biologically plausible and consistent with current understanding of psoriasis pathogenesis, no causal relationships can be inferred. Further studies, including functional and longitudinal analyses, are required to clarify the directionality and biological significance of these associations.

Collectively, our findings suggest a model in which host genetic architecture, particularly HLA-C-linked variants, shapes the immunological context, within which microbial communities may influence antigen presentation and local inflammation. Rather than initiating disease, microbial alterations may interact with genetically primed immune pathways to modulate disease expression.

Accordingly, psoriasis may be better viewed as a dynamic host–microbe ecosystem rather than a condition defined by uniform dysbiosis. Genotype-dependent immune differences may influence microbial composition already in non-lesional skin, with further remodeling occurring during lesion development.

Integrative host–microbiome approaches may, therefore, improve understanding of psoriasis heterogeneity and provide a framework for future studies exploring genotype-informed disease stratification, microbial biomarkers, and targeted therapeutic strategies.

## 4. Methods

### 4.1. Study Population

This study included a total of 77 healthy controls and 50 patients diagnosed with plaque-type psoriasis. However, DNA purification or PCR amplification failed for seven controls and three cases, excluding these individuals from this study. After inclusion in this study, one control patient was found to have inflammatory bowel disease and was, therefore, also excluded. For one control sample, sequencing failed. Sequencing failed for both non-lesional and lesional skin in seven cases, and in two additional cases, only non-lesional or lesional skin failed. Therefore, the final data analysis included 68 control samples and 39 samples from each non-lesional or lesional psoriatic skin (in total, representing 40 cases). The sample size was determined by the availability of well-characterized participants with matched lesional, non-lesional, and control skin samples, as well as corresponding genotype data. Given the complexity of multi-omics integration and the relatively high dimensionality of the data, this study was designed as an exploratory analysis aimed at identifying coordinated host–microbiome patterns rather than testing all possible pairwise associations.

None of the patients had used topical antiseptics, oral antibiotics, or systemic anti-inflammatory or immune-modulating treatment for at least three months prior to entering this study, and had not used topical corticosteroids on the target lesion two weeks prior to this study. Participants who were pregnant; had undergone tanning or intensive sun exposure in the past two weeks; were under the age of 18 years; had a known malignancy, psoriatic arthritis or other systemic inflammatory condition; or showed symptoms of infection at the time of sample collection were excluded from this study. All participants lived in the same geographical region of Sweden to reduce the impact of environmental and dietary factors. We analyzed the alpha- and beta-diversity of the microbiomes and correlated them with the different factors of the patients to see if there would be any potential confounding factor to run the integration, and did not find any significant correlation. Written informed consent was obtained from all participants, and this study was approved by the Ethics Committee of Linköping University, Linköping, Sweden (approval number: 2014/179-31). The participants’ gender, age, height, weight, current diseases and medications, smoking and alcohol habits, and family history of psoriasis were recorded. The severity of psoriasis was assessed using the Psoriasis Area and Severity Index (PASI) by a trained dermatology nurse.

### 4.2. Sample Collection, Preparation, and Sequencing

Samples were taken from lesional skin of the elbow and non-lesional skin at an adjacent cutaneous location at least 10 cm from the lesional skin in patients with psoriasis, and from the elbow skin in healthy controls. The samples were taken by swabbing a 4 × 4 cm area with a flocked swab soaked in 1 mL of liquid Amies (ESwab™, Copan Diagnostics Inc., Murrieta, CA, USA). Since colonization of bacteria in the skin depends on the skin site [55], all samples were taken from the elbow area. The samples were stored at −20 °C until DNA isolation.

Blood samples were also collected in EDTA from all included patients and stored at −80 °C prior to extraction.

### 4.3. Single-Nucleotide Polymorphisms (SNP) Selection

A total of 56 single-nucleotide polymorphisms (SNPs) were selected based on a combination of prior genetic association evidence and biological relevance to psoriasis pathogenesis. SNP selection was guided by a systematic literature review in PubMed and interrogation of the GWAS Catalog (https://www.ebi.ac.uk/gwas, accessed on 17 May 2023) to identify loci consistently associated with psoriasis susceptibility.

Priority was given to variants located in or near genes involved in key immunological pathways implicated in psoriasis, including antigen presentation (e.g., HLA-C region), cytokine signaling (e.g., IL-23/Th17 axis), innate immune responses, and epithelial barrier function. In addition, SNPs in candidate genes with established roles in inflammatory and immune-mediated processes were included to capture broader host pathways potentially relevant to host–microbiome interactions [27,28,29,49,56,57,58,59,60,61,62] (Supplementary Table S1). This strategy was intended to balance inclusion of well-established psoriasis susceptibility loci with biologically motivated candidate variants, thereby enabling exploration of coordinated host genetic and microbial patterns.

### 4.4. DNA Preparation

Genomic DNA was extracted from blood collected in EDTA using the MAGAttract DNA Blood Mini M48 Kit (Qiagen, Hilden, Germany), and DNA concentrations were determined by a NanoDrop ND-1000 (Thermo Fisher Scientific, Wilmington, DE, USA). Purified DNA was stored at −20 °C.

### 4.5. Genotype Assessment

Genotyping assays for 56 selected single-nucleotide polymorphisms (SNPs) were designed, validated and analyzed by the Mutation Analysis Core Facility (MAF) at the Karolinska University Hospital (Huddinge, Sweden; https://www.maf.ki.se/ accessed on 9 January 2022) using the MassARRAY system from Agena Bioscience (Agena Bioscience, San Diego, CA, USA). Assays were validated using human DNA samples from the CEU population, for which genotype data generated by the International HapMap Consortium are available. Reproducibility of the genotyping assays was ensured by repeated analysis on a number of samples. Further quality controls included twelve negative controls and twelve positive controls (CEU samples) on each 384-well plate, and the analyzed samples and SNPs were investigated for deviations from the Hardy–Weinberg equilibrium. Seven out of 56 SNP assays failed due to technical problems. Thus, 49 SNPs were included in the statistical analysis (Table S1).

### 4.6. Microbiome

The 16S microbiome data from 146 samples were processed with the nf-core:ampliseq (v 2.14.0) pipeline [63]. In brief, the raw sequencing reads were trimmed and filtered for quality, followed by generating ASVs and chimera removal using DADA2 [64]. These ASVs were then further filtered to remove non-bacterial and non-archaeal ASVs (potential sequencing or PCR artefacts) or of mitochondrial origin, followed by taxonomic assignment with SILVA (version 138.2) [65] using DADA2. Low-abundance ASVs were removed with the following filtering: only ASVs present in at least 10 samples with a minimum of 5 reads were retained. The filtered ASVs were then curated with LULU (v 0.1.0) [66] with the post-clustering method using VSEARCH (v 2.30.0) [67]. Taxonomic aggregation to species-level abundances was achieved using the phyloseq:tax_glom() function, which was then used for further downstream analyses. The QC analysis of the 16S data has been added as Supplementary Table S2. Prior to data integration, species representing <1% of the microbiome, as well as samples that were outliers in terms of their library size, were removed from the analysis before normalizing the data with the centered log-ratio using the ‘logratio.transfo()’ function from the R package mixOmics (v 6.18.1) [68].

### 4.7. Genotype Markers

The markers from the genotypic data (SNPs) were filtered based on a minimum call-rate of 85% and with a minimum allele frequency of 5%. These markers were then converted to a numeric dataset, and the distribution of the markers was visualized with PCA after scaling using FactoMineR (v 2.8) [69]. The significance of different sample variables like severity, gender, etc., on the distribution of the markers was calculated with PERMANOVA using the ‘adonis2()’ function with the parameter ‘permutations = 999′ from the R package Vegan (version 2.6-4) (https://CRAN.R-project.org/package=vegan, accessed on 7 April 2025). Allele dosage was analyzed with respect to the risk allele for psoriasis, and the alternate allele served as the reference allele, as defined in the GWAS Catalog (https://www.ebi.ac.uk/gwas, accessed on 17 May 2023), and if the SNP in question was not a GWAS locus for psoriasis, then the dosage was calculated with respect to the alternate allele, as defined by the NCBI dbSNP (https://www.ncbi.nlm.nih.gov/snp, accessed on 17 May 2023).

### 4.8. Multi-Omics Integration

The supervised multi-omics method DIABLO [70], implemented as the block.splsda() function in the mixOmics package, was used to integrate the original SNP and species data blocks in order to construct latent components for the prediction of categorical outcomes (either lesional or non-lesional psoriatic skin vs. healthy controls).

For the final model, the optimal number of latent components was calculated using the perf() function, and the optimal number of original variables per data block and latent component was computed using the tune.block.splsda() function. The results were visualized using functions in the mixOmics R package. The code used to select and tune the parameters and also to assess the performance of the model, including the variable selection stability, is included in the scripts that are part of the GitHub repository. The prediction capability of the model, including the AUC curves, is also included in the scripts for the different integrations.

## Figures and Tables

**Table 1:** Patients’ demographic and clinical characteristics.

	Cases	Controls
Number of subjects	37	65
Age, years, mean ± SD ^a^ (range)	53.4 ± 14.5 (24–76)	48.9 ± 16.1 (19–86)
Male:female ratio, n	18:19	20:45
Body mass index, mean ± SD (range)	26.7 ± 3.1 (20.8–33.0)	24.4 ± 2.8 (18.8–31.7)
PASI, mean ± SD (range)	6.2 ± 4.6 (0.5–25)	
Family history of psoriasis, %	72.9	6.2
Smoker, %	10.5	3.1
Alcohol intake ≥ 2 times/month, %	15.8	12.3

^a^ Standard deviation.

## Data Availability

The analytical scripts and QC analysis of the 16S data are available in the following public repository: https://github.com/NBISweden/Support_7993, accessed on 2 April 2026.

## Supplementary Materials

The following supporting information can be downloaded at https://www.mdpi.com/article/10.3390/ijms27094116/s1.
