# Supplementary material for: Integrated Host Genetics and Skin Microbiome Profiling Suggest an HLA-C–Peptostreptococcus Axis in Psoriasis
Source: Int J Mol Sci. 2026 May 4;27(9):4116. doi: 10.3390/ijms27094116 (PMC13163479; doi:10.3390/ijms27094116)
Supplement: Supplementary file 1 [file ijms-27-04116-s001.zip › Suppl-Table S1.pdf]

Supplementary Table S1. The study population's allele frequencies for single nucleotide polymorphisms (SNPs), and genes located at each locus. Bold marked allele used for allele dosage analysis.

SNP ID	A1[a]	A2[b]	Genes[c]	Reference
rs1012356	**A=0.369**	T=0.631	IL22	1
rs10484554	**T=0.325**	C=0.675	HLA-C, HLA-B	2
rs1056198	**C=0.481**	T=0.519	RNF114	3
rs1076160	**T=0.475**	C=0.525	TSC1	4
rs10782001	**G=0.319**	A=0.681	FBXL19	5
rs10789285	**G=0.329**	T=0.671	LRRC7	6
rs10865331	**A=0.383**	G=0.617	B3GNT2	3
rs10979182	G=0.363	**A=0.637**	KLF4	3
rs11121129	**A=0.364**	G=0.636	SLC45A1, TNFRSF9	3
rs1131014[e]			HLA-C[d]	-
rs11652075	T=0.396	**C=0.604**	CARD14	3
rs11795343	C=0.356	**T=0.644**	DDX58	3
rs12191877	**T=0.279**	C=0.721	HLA-C	4
rs12445568	**C=0.35**	T=0.65	PRSS53, FBXL19	3
rs1250546	G=0.409	**A=0.591**	ZMIZ1	3
rs1265181	**C=0.256**	G=0.744	HCG27	7
rs12924903	**A=0.319**	G=0.681	FBXL19	5
rs1295685	A=0.181	**G=0.819**	IL13, IL4	3
rs1530551	T=0.438	**C=0.563**	IL36RN[d]	-
rs2046068	**G=0.269**	T=0.731	IL22	1
rs2145623	C=0.281	**G=0.719**	NFKB1A	8
rs2201841	**G=0.266**	A=0.734	IL23R	4
rsrs2227314[e]			IL12A	9
rs2243135	**C=0.438**	G=0.563	IL12A[d]	-
rs2278717[e]			IL36RN[d]	-
rs2451258	**C=0.377**	T=0.623	TAGAP	3
rs2485558	**G=0.286**	C=0.714	RYR2	8
rs2546893	G=0.494	**A=0.506**	IL12B[d]	-
rs2569253	T=0.481	**C=0.519**	IL12B[d]	-
rs2675662	G=0.435	**A=0.565**	CAMK2G[d]	-
rs2700987	C=0.409	**A=0.591**	ELMO1	3
rs27432	**A=0.396**	G=0.604	ERAP1	3
rs27524	**A=0.431**	G=0.569	ERAP1	10
rs2853694	T=0.435	**G=0.565**	IL12B[d]	-
rs3207561[e]			HLA-C[d]	-
rs3802826	G=0.481	**A=0.519**	ETS1	3
rs4406273	**A=0.227**	G=0.773	HLA-B, HLA-C	3
rs4561177	G=0.425	**A=0.575**	ZC3H12C	3
rs4795067	**G=0.435**	A=0.565	NOS2	5
rs4819554	G=0.195	**A=0.805**	IL17RA	11

rs495337	**G=0.481**	A=0.519	RNF114	5
rs582757	**C=0.377**	T=0.623	TNFAIP3	3
rs610604	**G=0.403**	T=0.597	TNFAIP3	4
rs62149416	C=0.429	**T=0.571**	FLJ16341, REL	3
rs645078	C=0.351	**A=0.649**	RPS6KA4, PRDX5	3
rs6677595	C=0.287	**T=0.712**	LCE3B, LCE3	3
rs6927172[e]			TNFAIP3[d]	-
rs702873[e]			REL	10
rs72916862	**C=0.188**	T=0.813	IL17A[d]	-
rs730691	T=0.383	**C=0.617**	IL12B[d]	-
rs7536201[e]			RUNX3	3
rs7615589	**A=0.319**	G=0.681	IL12A[d]	-
rs8016947	T=0.463	**G=0.538**	NFKBIA	10
rs892085	G=0.325	**A=0.675**	ILF3, CARM1	3
rs9504361	G=0.431	**A=0.569**	EXOC2, IRF4	3
rs963986	**C=0.188**	G=0.812	PTRF, STAT3, STAT5A/B	3

[a] Minor allele frequency.

[b] Major allele frequency.

[c] Genes cited for each locus were based on genes mentioned in the original studies identifying the susceptibility variants.

[d] SNPs not previously studied in psoriasis and, selected based on suspected immunological relevance in psoriasis.

[e] Excluded from study since assay failed.

References

1. Weger W, Hofer A, Wolf P, El-Shabrawi Y, Renner W, Kerl H, et al. Common polymorphisms in the interleukin-22 gene are not associated with chronic plaque psoriasis. Experimental dermatology. 2009;18(9):796-8.
2. Liu Y, Helms C, Liao W, Zaba LC, Duan S, Gardner J, et al. A genome-wide association study of psoriasis and psoriatic arthritis identifies new disease loci. PLoS genetics. 2008;4(3):e1000041.
3. Tsoi LC, Spain SL, Knight J, Ellinghaus E, Stuart PE, Capon F, et al. Identification of 15 new psoriasis susceptibility loci highlights the role of innate immunity. Nature genetics. 2012;44(12):1341-8.
4. Nair RP, Duffin KC, Helms C, Ding J, Stuart PE, Goldgar D, et al. Genome-wide scan reveals association of psoriasis with IL-23 and NF-kappaB pathways. Nature genetics. 2009;41(2):199-204.
5. Stuart PE, Nair RP, Ellinghaus E, Ding J, Tejasvi T, Gudjonsson JE, et al. Genome-wide association analysis identifies three psoriasis susceptibility loci. Nature genetics. 2010;42(11):1000-4.
6. Biswas S, Pal S, Majumder PP, Bhattacharjee S. A framework for pathway knowledge driven prioritization in genome-wide association studies. Genetic epidemiology. 2020;44(8):841-53.
7. Villarreal-Martínez A, Gallardo-Blanco H, Cerda-Flores R, Torres-Muñoz I, Gómez-Flores M, Salas-Alanís J, et al. Candidate gene polymorphisms and risk of psoriasis: A pilot study. Experimental and therapeutic medicine. 2016;11(4):1217-22.

8. Ellinghaus E, Ellinghaus D, Stuart PE, Nair RP, Debrus S, Raelson JV, et al. Genome-wide association study identifies a psoriasis susceptibility locus at TRAF3IP2. Nature genetics. 2010;42(11):991-5.
9. Cargill M, Schrodi SJ, Chang M, Garcia VE, Brandon R, Callis KP, et al. A large-scale genetic association study confirms IL12B and leads to the identification of IL23R as psoriasis-risk genes. American journal of human genetics. 2007;80(2):273-90.
10. Strange A, Capon F, Spencer CC, Knight J, Weale ME, Allen MH, et al. A genome-wide association study identifies new psoriasis susceptibility loci and an interaction between HLA-C and ERAP1. Nature genetics. 2010;42(11):985-90.
11. Batalla A, Coto E, González-Lara L, González-Fernández D, Gómez J, Aranguren TF, et al. Association between single nucleotide polymorphisms IL17RA rs4819554 and IL17E rs79877597 and Psoriasis in a Spanish cohort. Journal of dermatological science. 2015;80(2):111-5.
